# Is this study feasible? Facilitating management of pragmatic trial planning milestones under a phased award funding mechanism

**DOI:** 10.1186/s13063-019-3387-3

**Published:** 2019-05-30

**Authors:** Paula Darby Lipman, Leanora Dluzak, Catherine M. Stoney

**Affiliations:** 1 Westat, 1600 Research Boulevard, Rockville, MD 20850 USA; 2 National Heart, Lung, and Blood Institute (NHLBI), National Institutes of Health (NIH), 6701 Rockledge Drive, Bethesda, MD 20817 USA

**Keywords:** Pragmatic trials, Phased award, Trial management, Trial conduct, Milestones, Metrics, Improving efficiencies

## Abstract

**Background:**

Improving efficiencies in clinical research is crucial to translation of findings into practice and delivery of effective, patient-centered health care. This paper describes a project that monitored pragmatic clinical trials by working with investigators to track achievement of early phase milestones. The National Institutes of Health (NIH) Pragmatic Trials Collaborative Project supported scientifically diverse, low-cost, randomized, controlled, pragmatic clinical intervention trials. Funds were available through a cooperative agreement award mechanism, with the initial phase supporting trial planning and the subsequent 4-year awards funding trial implementation. A coordinating center provided evaluation and administrative support, which included capturing progress toward achieving milestones.

**Methods:**

Six funded trials participated in monthly calls throughout the first year to identify and demonstrate metrics and deliverables for each milestone in the Notice of Grant Award. Interviews were conducted with investigators, trial team members, and NIH program officers/project scientists to discuss their perceptions of the impact and value of the management strategy.

**Results:**

Five of six trials transitioned to the implementation phase with milestones ranging from 6 to 15 and quantifiable metrics ranging from 15 to 33, for a total of 121 deliverables. One third of the metrics (42, 35%) were trial-specific. Trial teams reported that the oversight was onerous but complemented their management strategies; program officers/project scientists found that documentation submitted for review was sufficient to assess trial feasibility; and investigators reported advantages to the phased award mechanism, such as leverage to secure commitments from stakeholders and collaborators, help with task prioritization, and earlier consultation with key members of the trial team.

**Conclusions:**

Implementing systematic approaches to identify milestones and track metrics can strengthen the evidence base regarding time and effort to plan and conduct pragmatic clinical trials. Investigators were unaccustomed to producing evidence of performance, and it was challenging to determine what documentation to provide. Efforts to standardize expectations regarding milestones that mark a significant change or stage in trial development or that represent minimum success criteria may provide guidance for more effective and efficient trial management. A framework with clearly specified metrics is especially critical for transparency, particularly when funding decisions are contingent on both merit and feasibility.

## Background

Improving efficiencies across all phases and types of clinical research is crucial to accelerating translation of findings into practice, leading to better delivery of effective, patient-centered care [1–3]. The complexities of conducting clinical trials are well known [4, 5], and numerous strategies at multiple levels have been proposed or adopted to address challenges with research design and conduct [6]. This paper describes outcomes of a unique National Institutes of Health (NIH) project that provided management and coordination support for a set of pragmatic clinical trials (RFA-HL-14-019) by working closely with principal investigators (PIs) during the early phase of the trial to identify and track achievement of explicit trial planning milestones.

The NIH Pragmatic Trials Collaborative Project, initiated in 2014 to support scientifically diverse, low-cost, patient-centered, randomized, controlled, pragmatic clinical intervention trials, incorporated several strategies to ensure optimal trial planning and conduct and to promote early identification of potential threats to trial success [7]. The first is the use of cooperative agreements, wherein NIH program officers (POs) and project scientists (PSs) work jointly with the PIs to serve as a resource and provide scientific guidance throughout the life cycle of the trial. Under this cooperative agreement, members of the project participated in joint activities to gain a better understanding of the struggles and successes of trial planning, explore the significance of stakeholder engagement and other factors, and anticipate potential challenges to meeting patient accrual and data management objectives.

**Fig. 1:**
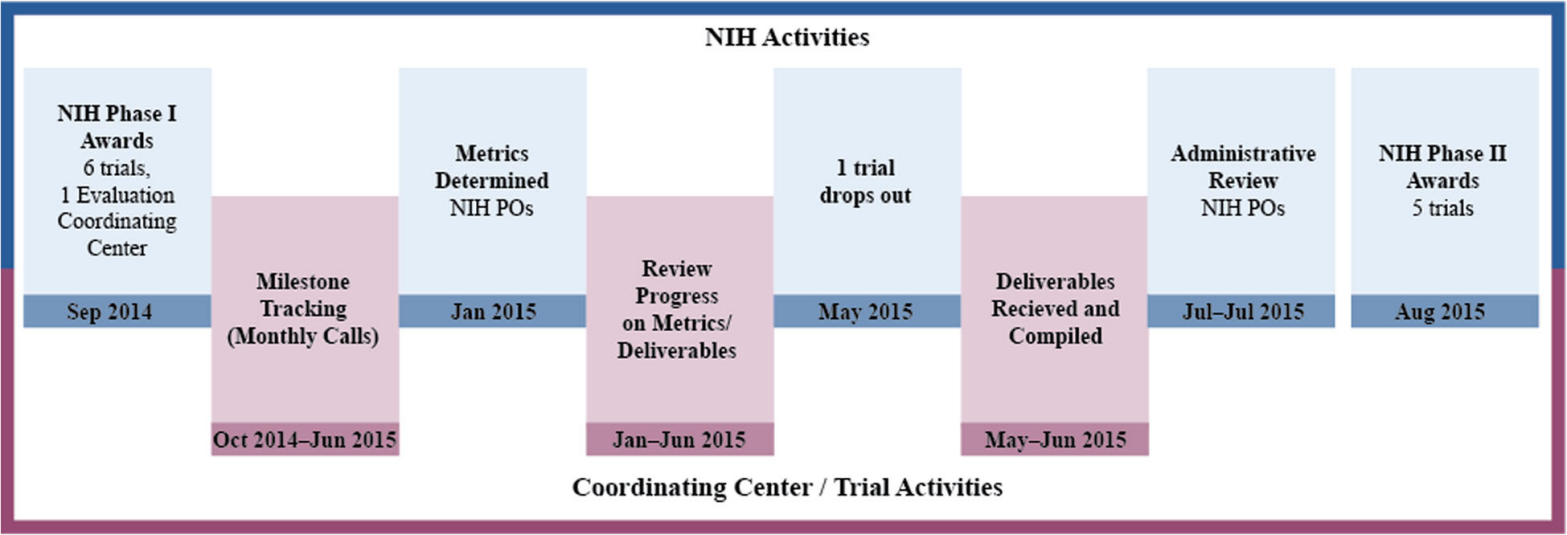
Planning phase: process flowchart

The second strategy to enhance the likelihood of trial success is the phased award mechanism, increasingly used across NIH in recent years, which incorporates processes to identify early phase (i.e., first year) milestones and trials at risk. Funds for the trial implementation phase (i.e., subsequent 4 years) are contingent on administrative review of milestone achievement. Milestones are the qualitative benchmarks of accomplishment of essential goals, and most require a sequence of steps that collectively represent milestone achievement. All trials were required to complete the identified planning milestones within the early phase time period. The milestones reflect the critical start-up steps as delineated in the application, and are incorporated in the Notice of Grant Award (NoGA). The NIH has utilized various approaches to the phased award mechanism, including varying the length of time allotted to complete the planning milestones. Under the NIH Pragmatic Trials Collaborative Project described in this paper, activities expected to be accomplished in the early phase (approximately 12 months) included refinement of existing resources, further development of study partnerships, and finalization of trial protocols. There were sufficient funds for full implementation of all trials. To advance the sponsor’s interest in pragmatic trial designs and determine whether they can help to bridge the translation gap, a companion award (RFA-HL-14-020) was made to a coordinating center (awarded to Westat, an employee-owned research organization headquartered in Rockville, MD, USA) to evaluate the funded trials from a process and operational view, particularly during the planning phase, which included assembling appropriate documentation for the administrative review conducted by the NIH [8]. Figure 1 illustrates this process and timeline.

Pragmatic trials conducted in real-world settings have design features that distinguish them from more explanatory trials [9–16]. Given the likelihood of additional unanticipated challenges that may be encountered as investigators secure buy-in from stakeholders and gatekeepers, test feasibility of systems and data collection methods pertaining to primary outcomes, and confirm availability of patient populations, understanding the management of critical start-up activities in the early phase of more pragmatic trials may be especially relevant to trial designers, sponsors, and research partners [17–20]. While considerable research exists on milestones or features associated with traditional clinical trials [21, 22], only recently have efforts been undertaken to systemically capture critical factors, contingencies, and timelines associated with trial planning for more pragmatic research [23]. Furthermore, the trials funded under this specific initiative had the additional requirement of a lower cost budget compared to many other funding opportunities, such that factors related to management efficiency, workflow, and resource utilization were even more critical [19]. With the support and cooperation of the awardees and their POs, the additional management support provided by the coordinating center facilitated a learning and collaborative platform and offered an opportunity to capture and share lessons learned regarding identifying evidence of achievement of planning milestones under this phased award.

## Methods

Based on the available literature on clinical trial milestones and requirements of the funding announcement, the coordinating center developed a general framework to categorize milestones as Collaborations, Materials and Methods, Clearances, Study Population, Resources, and Patient Information Management. The framework (Table 1) was used to align milestones for each trial, with those appearing to fall outside these categories classified as Trial-Specific.

**Table 1:** Pragmatic trial planning: general framework for milestones and metrics

Milestones	Illustrative planning phase metrics
A. Collaborations	
Develop and document collaborations and partnerships	1. Copies of Memo of Understanding or contracts with organizations involved in the clinical trial 2. Copies of signed agreements of clinical sites (if applicable) 3. Copies of letters of support from study champions/investigators in relevant delivery locations 4. Copies of executed data use agreements
B. Materials and Methods	
Finalize protocol, manual(s) of procedures, data collection forms	1. Copy of Institutional Review Board (IRB)-approved protocol 2. Copy of final manual of procedures 3. Copies of IRB-approved data collection forms (if applicable), including methods for data management and data quality 4. Validated methods to collect data from existing electronic sources (if applicable) 5. Demonstration of methods for integration of data collection forms into electronic resources (if applicable)
C. Clearances	
Achieve necessary human subject protection approvals and procedures	1. Copy of IRB approval to conduct clinical trial 2. Copy of IRB-approved informed consent (if applicable) 3. List of Data and Safety Monitoring Board (DSMB) members and copy of DSMB charter 4. Final IRB-approved data and safety monitoring plan
D. Study Population	
Evidence of adequate potential study population	1. Environmental survey of potential study participants (with estimates by site and plan for tracking progress) 2. Recruitment plan with recruitment milestones, alternative strategies, and participant invitation (if applicable)
E. Patient Information Management	
Patient information management	1. Evidence of feasibility of data collection materials, sources, and processes 2. Documentation of methods for adding patient follow-up information to the trial database
F. Resources	
Equipment, staff, training, and budget	1. Documentation of equipment requisitions, availability of adequate and appropriate staff, and budget in line with trial needs 2. Documentation of training needs and training curriculum
G. Trial-Specific	

Six awards were made under this initiative. As is customary under this phased approach, the NIH PO assigned to each trial worked with the PI to finalize the planning milestones (September 2014) and subsequently to determine specific metrics associated with each (January 2015). The lag in time was due to the awareness by the project leadership that the indicators needed for the administrative review were at the metric rather than the milestone level. Metrics provide objectively measurable evidence of milestone progress, overall functioning of the trial, and forewarnings about factors that need attention. Tracking achievement on these performance metrics was intended to encourage improvement, increase effectiveness, and manage expectations.

### Management support

From December 2014 (introductory kick-off meeting) through June of 2015, each PI and members of their trial teams participated in recorded monthly conference calls with the coordinating center to discuss progress. These were collaborative 1-h discussions of task prioritization, alignment of metrics with milestones, estimates of completion dates, and negotiation on the type of deliverable to be provided (e.g., screen shots, lists of variables found in data dictionaries, copies of signed agreements). Acceptable forms of documentation included PDFs, Word documents, or Excel files. A tailored tracking form, developed for each trial and updated and redistributed following each monthly call, was used to capture information including date of completion, type of deliverable, and deliverable receipt date. One 2-day in-person meeting was also held toward the end of the first year.

A process was developed to package and deliver documents to the NIH for their internal administrative panel review. Performance documentation was collected using a secure File Transfer Protocol (FTP) server. Submission instructions were provided to the trial teams and included a document naming convention to identify documents and maintain version control as they were received via the FTP server. The coordinating center conducted an adequacy check as materials were collected and worked in collaboration with the trial teams if questions arose. The coordinating center did not assess the documentation on scientific merit but from an operational point of view. A binder for each trial was compiled and included a one-page summary; all the documentation received; and a Reviewer Checklist that itemized each deliverable, provided a column for optional reviewer comments, and requested indication of a satisfactory assessment for each metric. Hard copies of the binders and a flash drive with all documentation were delivered to the NIH where two independent NIH POs (i.e., not the PO of the grant) served as reviewers and provided recommendations for continued funding.

### Evaluation

Recordings and meeting minutes from the 1-h monthly calls, proceedings of the annual 2-day in-person meeting, and semi-structured interviews were the qualitative data for the evaluation. A semi-structured guide was developed for the interviews conducted with PIs and trial team members (August through September 2015) and the NIH POs/PSs (October 2015) on their perceptions of the impact and value of the management strategy. Data were analyzed by the coordinating center (PDL and LD) using a modified grounded approach [24], with recordings accessed for clarity or to supplement meeting minutes and notes.

## Results

During the early phase of funding, one of the investigators recognized that assumptions about eligibility criteria and availability of patients were flawed, leading to withdrawal prior to the administrative review to pursue a more appropriate funding mechanism. Based on recommendations from the NIH internal administrative review, the remaining five trials [25–29] were approved for implementation funding (summarized in Table 2).

**Table 2:** Funded trials (Phase II)

Trial name	PI (Affiliation)/Sponsor	Trial title	Significance
ENGAGES	M. Avidan, MD (Washington University)/National Institute on Aging (NIA)	Electro-encephalograph Guidance of Anesthesia to Alleviate Geriatric Syndromes	Reduce post-operative delirium associated with cognitive impairment and falls
HUSH	D. Buysse, MD (University of Pittsburgh)/National Heart, Lung, and Blood Institute (NHLBI)	Pragmatic Trial of Behavioral Interventions for Insomnia in Hypertensive Patients	Reduce insomnia disorder using non-drug treatment in primary care
PART	H. WANG, MD (University of Texas at Houston; formerly University of Alabama at Birmingham) / NHLBI	Pragmatic Trial of Airway Management in Out-of-Hospital Cardiac Arrest	Identification of best approach for out-of-hospital cardio-pulmonary arrest
PROOFCheck	M. Gong, MD; O. Gajic, MD (Albert Einstein College of Medicine of Yeshiva University)/NHLBI	Prevention of Severe Acute Respiratory Failure in Patients with PROOFCheck	Prevent acute respiratory failure leading to organ failure
REDAPS	S. Halpern, MD (University of Pennsylvania)/NIA	Default Palliative Care Consultation for Seriously Ill Hospitalized Patients	Determine effectiveness and cost of inpatient palliative care consult services

The number of milestones delineated in the five award notices (NoGAs) ranged from 6 to 15. Most milestones had one or more associated metrics; the total number of metrics ranged from 15 to 33. One third of the metrics (42, 35%) were associated with trial-specific milestones. Metrics, rather than milestones, are presented in Table 3, as these were the explicit indicators by which performance and progress were assessed.

**Table 3:** Planning phase: number of metrics by trial

	Trial 1	Trial 2	Trial 3	Trial 4	Trial 5	Total metrics
A. Collaborations	2	4	3	5	3	17
B. Materials and Methods	4	8	5	7	4	28
C. Clearances	4	5	2	4	2	17
D. Study Population	2	2	1	2	1	8
E. Patient Information Management	1	0	2	1	1	5
F. Resources	0	1	0	1	2	4
G. Trial-Specific	6	8	20	6	2	42
TOTAL	19	28	33	26	15	121

### Specification of deliverables

A common challenge in discussions with the trial teams was specifying the deliverable or documentation associated with each metric; this was particularly evident for those that were more unique (e.g., trial-specific) or that represented technological or system-level progress. Occasionally the same deliverable was linked to more than one metric, and this was clearly documented on the tracking form and in the administrative review materials for the NIH. All of these issues were resolved through discussion of options and clear communication with the PIs about expectations.

Table 4 provides a list of trial-specific metrics and a description of their deliverables, further grouped as related to Training (of research staff or interventionists); Stakeholder buy-in or partner engagement; Data management; Intervention refinement and finalization; Recruitment/accrual feasibility; and Information technology (IT) or systems interoperability. Testing feasibility of systems, ensuring buy-in from stakeholders, and assessing intervention acceptability were among the critical achievements required in the planning phase.

**Table 4:** Illustrative planning phase trial-specific metrics and deliverables

Trial-specific metric	Description of deliverable
Training	
Train staff on assessment methods	Summary of training with staff
Train staff on protocol	Summary of training with staff on the protocol
Training sessions completed in intervention settings	Table of trainings completed and planned for future
Stakeholders/partner engagement	
Assessment of provider satisfaction with interventions	Satisfaction survey items and feedback based on data from five physicians
Identification of local champions	List of local champions and their letters of support
Data management	
Determine feasibility of obtaining baseline and 30-day follow-up data	Obtained 30-day follow-up data on 85% of patients enrolled in pilot
Validate capture of all proposed outcomes in a sample of de-identified patients	Description of process to finalize list of outcomes
Data form development completion	Copy of data collection forms
Intervention	
Create preliminary versions of educational materials	Preliminary draft of educational materials
Final adjustments made to interventions	Procedures manual
Recruitment/accrual feasibility	
Enroll (subset) of pilot study cohort	Consort diagram summarizing pilot recruitment
Testing and validation in validation cohort: test candidate model with summary of findings in derivation cohort	Application of model for patients with and without an event of concern
Information technology/systems	
Fully functional research recruitment alert in electronic health records	Screenshot of pop-up alert as evidence that system is functional
Online intervention ready for deployment	Screen shots of login and modules
Web-based data collection procedures in place	Screen shots to capture screening data
Electronic health records data pull methods tested and validated	Three sample Subject Data Tables
Web conferencing capabilities for intervention	Minimum technical and support requirements
Development of strategy to automate intervention into electronic medical records	Flow diagram illustrating how intervention is integrated with electronic medical records and other clinical data systems
Develop patient identification and automatic ordering processes in the electronic health records	List of patient eligibility criteria screening; specifications for automated order

Table 4 illustrates that many metrics categorized as trial-specific will test assumptions regarding patient recruitment or accrual, intervention delivery, and management of outcome data, requiring that the associated deliverables demonstrate achievements pertaining to access to electronic health records or functioning of IT and database systems. Descriptions of each deliverable were included in the summary reports provided to reviewers.

### Qualitative findings

Analysis of qualitative data sources, including monthly meeting minutes, the annual in-person meeting transcript, and semi-structured qualitative interviews, indicated that members of the trial teams found the oversight process onerous at times but reflected that it mostly improved or complemented their own management strategies. POs/PSs benefited from enhanced engagement with the PIs and the opportunity to learn more about pragmatic trial management and implementation of the phased award mechanism, and reported that the extensive documentation submitted by the coordinating center provided sufficient evidence to assess trial feasibility. Overall, the PIs reported several distinct advantages of the phased award mechanism, including how pressure to demonstrate progress helped to prioritize essential project management tasks, led to earlier engagement with technical and data management staff, and provided additional leverage to secure commitments from external stakeholders and collaborators. Table 5 provides a summary of themes identified in the interviews conducted during the first project year with PIs and POs/PSs, and Table 6 includes select quotes that capture these sentiments.

**Table 5:** Summary of Themes from the Planning Phase with PIs, Research Teams, and POs/PSs

Oversight (Planning Phase)	
PI/Trial Team	• Westat and the use of milestones helped with accountability • Helped the grantees define the milestones • Encouraged flexibility in interpreting milestones • Providing documentation was extra work • Did not have a real need for Westat • Westat gave unclear guidance
POs/PSs	• Westat’s role relative to the POs was not always clear • Input from Westat on grantee milestones provided important clarity to help NIH make decision about funding • Assistance provided by Westat enabled POs to focus on the science • Independent review process was very well organized
Value of in-person conference	
PI/Trial Team; POs/PSs	• Everyone who attended the meeting enjoyed it, found it helpful • Useful to meet other grantees/hear about their trials • Felt like a community of pragmatic trialists • Useful to hear the NIH perspective
POs/PSs	• Useful to hear common description of what pragmatic trials are; everyone on same page
Phased award mechanism	
PI/Trial Team; POs/PSs	• Planning phase helpful in showing what works, where problem areas are and what is needed to succeed; good for pilot and feasibility data
PI/Trial Team	• Good, more efficient mechanism for clinical trials; studies with many unknowns • A way for NIH to be cost-effective • Provides structure to inexperienced PIs • Keeps the pace of work up, accountability • Provides more honest relationship with funding agency
POs/PSs	• Significant involvement of NIH in all decision-making • Mechanism allowed for PO to serve as liaison between trial team and administrative leadership • Number of milestones should be comparable across grantees (e.g., no more than 15-20)

**Table 6:** Illustrative quotes from participants in monthly calls and year 1 in-person project meeting

Theme	Comment
Value of oversight (planning phase)	“… this process is very helpful. It’s making us think more carefully about our work … and think about the big picture and where we might be weak.”
	“If you were just to dive into a busy clinical trial, I think you would cut corners and miss some of those important questions.”
	“… it paid off in being confident that we could do these things for the long term … we felt comfortable proceeding with the next iteration.”
	“You’re highly motivated and incentivized to hit those milestones because you know this is a go/no-go. And some of them you have anxiety about because they’re beyond your control.”
	“We had in mind that there were very specific milestones and the evaluation on a monthly basis was helping to keeping *[sic]* us on track.”
	“I like the planning year. It gave us more time to devote to having a good plan, which ultimately saves time down the road.”
	“The project management aspect that Westat provided was useful … in that way a best practice of research management.”
Positive aspects of pressure to meet milestones	“It helped me in terms of my subcontract sites and my IT.”
	“When we showed [stakeholders] we were falling off track in any of these areas, it very much focuses us on working on that problem specifically.”
	“It makes you get started right away, which is a good thing.”
	“I met with my stakeholders and got their commitment; that was part of the planning year but it wasn’t [directly] for patient recruitment.”
	“We were working with a system we hadn’t worked with before that didn’t have much research infrastructure … it was relationship building with the leaders and the informatics team …”
	“… helpful to align institutional leadership.”
Phased award mechanism	“… even though I know I hit all of the milestones, this was a new mechanism for me [so I didn’t necessarily have confidence re: implementation funds].”
	“This is new for many of us … and it sounds like it actually may be relatively new for some of the agencies as well.”
	“One of the things I would include would be budgeting [such that] the pilot year could really be a year. Truncating that does apply more pressure.”
	“… [helpful to] work with the program official [if] milestones are a bit overly ambitious [for the planning year].”

## Discussion

The observations from this project have the potential to improve the knowledge base regarding macro-level strategies to increase clinical research productivity, thereby demonstrating responsible stewardship of publicly funded science. As this was a unique design under a specific NIH solicitation with a small number of low-cost trials, future efforts are needed to expand upon our preliminary findings, for example by assessing the association between planning milestones and successful participant recruitment or accrual. However, this effort achieved one of its overarching intentions — early identification of an at-risk trial — as one of the Phase I awardees discovered during this phase that the patient population in their single-site trial was insufficient. Other positive elements included co-management of the planning process, support for generating reliable metrics to assess progress, and a collaborative environment that provided a forum for investigators to share their progress with other researchers in different fields and to communicate in person with their NIH POs/PSs.

Synthesis of lessons from strategies for early identification of trial risk factors can contribute to management guidance and standardization [21], potentially of benefit to both trial designers and funding organizations. Results from our efforts to systematically categorize critical start-up milestones illustrate the need for additional research in this area [30]. The approach used to differentiate milestones specific to the trial from those more likely to be common across all trials suggests that this distinction is not clear-cut. We also speculated but could not confirm whether the relatively large proportion of trial-specific milestones reflects something unique to more pragmatic trials with particular constraints due to their conduct in real-world settings. However, given the overall failure of many trials to meet recruitment or dissemination goals [31, 32], there is value in efforts such as the phased award mechanism to identify critical precursors demonstrating a potential study population adequate to meet the sample size of the trial, and other factors associated with trial feasibility and effective resource utilization.

## Conclusions

Strategies such as cooperative agreements and phased mechanisms are increasingly being adopted and integrated into biomedical funding practices. From the perspective of the investigator, advantages of the phased mechanism include clear delineation of the development time period, as well as specification of critical milestones to be accomplished, which helps prioritize task management, galvanize gatekeepers, and emphasize feasibility testing [33]. The funding institute benefits, as their investment in the trial is potentially less risky, with a clearly delineated process for internal review and clear stopping rules. The methodology developed and implemented by the coordinating center to facilitate management of early phase progress has been disseminated and adapted for similar projects within the NIH.

With regard to the field of pragmatic research more generally, implementing systematic approaches to identify milestones and track metrics can strengthen the evidence base regarding the time and effort required to efficiently conduct and manage large simple trials [4], and this process has been proposed among a set of solutions to improve community-engaged implementation research [34] and the efficiency and effectiveness of clinical trial recruitment planning [35]. Although each awardee in this project was required to provide evidence of completion of metrics, there was considerable variability in number and type required. Future efforts to link early phase management support with trial implementation outcomes can support guidance regarding when flexibility and adaptation versus more rigid adherence to pre-determined milestones is appropriate [13]. Developing and disseminating a classification or framework to guide trial design and review is especially critical for transparency, particularly when funding decisions are contingent on both merit and feasibility [2].

## Abbreviations

DSMB: Data and Safety Monitoring Board
ENGAGES: Electro-encephalograph Guidance of Anesthesia to Alleviate Geriatric Syndromes (trial)
FTP: File Transfer Protocol
HUSH: Pragmatic Trial of Behavioral Interventions for Insomnia in Hypertensive Patients
IRB: Institutional Review Board
IT: Information technology
NHLBI: National Heart, Lung, and Blood Institute
NIH: National Institutes of Health
NoGA: Notice of Grant Award
PART: Pragmatic Trial of Airway Management in Out-of-Hospital Cardiac Arrest
PI: Principal investigator
PO: Program officer
PROOFCheck: Prevention of Severe Acute Respiratory Failure in Patients with PROOFCheck (Electronic Checklist to Prevent Organ Failure)
PS: Project scientist
REDAPS: Default Palliative Care Consultation for Seriously Ill Hospitalized Patients
RFA: Request for Applications
